# Comparing the Proteomic Profiles of Extracellular Vesicles Isolated using Different Methods from Long-term Stored Plasma Samples

**DOI:** 10.1186/s12575-024-00243-4

**Published:** 2024-06-19

**Authors:** Ana Torres, Lorena Bernardo, Carmen Sánchez, Esperanza Morato, Jose Carlos Solana, Eugenia Carrillo

**Affiliations:** 1 WHO Collaborating Centre for Leishmaniasis, Spanish National Centre for Microbiology, Instituto de Salud Carlos III, Majadahonda, Spain; 2 Centro de Investigación Biomédica en Red de Enfermedades Infecciosas, Instituto de Salud Carlos III, Madrid, Spain; 3 Proteomics Facility, Centro de Biología Molecular Severo Ochoa, Consejo Superior de Investigaciones Científicas, Universidad Autónoma de Madrid, Madrid, Spain

**Keywords:** Extracellular Vesicles, Ultracentrifugation, Size Exclusion Chromatography, Proteomics, Plasma

## Abstract

**Background:**

The lack of standardized protocols for isolating extracellular vesicles (EVs), especially from biobank-stored blood plasma, translates to limitations for the study of new biomarkers. This study examines whether a combination of current isolation methods could enhance the specificity and purity of isolated EVs for diagnosis and personalized medicine purposes.

**Results:**

EVs were isolated from healthy human plasma stored for one year by ultracentrifugation (UC), size exclusion chromatography (SEC), or SEC and UC combined (SEC + UC). The EV isolates were then characterized by transmission electron microscopy imaging, nanoparticle tracking analysis (NTA) and western blotting. Proteomic procedures were used to analyze protein contents. The presence of EV markers in all isolates was confirmed by western blotting yet this analysis revealed higher albumin expression in EVs-UC, suggesting plasma protein contamination. Proteomic analysis identified 542 proteins, SEC + UC yielding the most complex proteome at 364 proteins. Through gene ontology enrichment, we observed differences in the cellular components of EVs and plasma in that SEC + UC isolates featured higher proportions of EV proteins than those derived from the other two methods. Analysis of proteins unique to each isolation method served to identify 181 unique proteins for the combined approach, including those normally appearing in low concentrations in plasma. This indicates that with this combined method, it is possible to detect less abundant plasma proteins by proteomics in the resultant isolates.

**Conclusions:**

Our findings reveal that the SEC + UC approach yields highly pure and diverse EVs suitable for comprehensive proteomic analysis with applications for the detection of new biomarkers in biobank-stored plasma samples.

**Supplementary Information:**

The online version contains supplementary material available at 10.1186/s12575-024-00243-4.

## Background

Blood is one of the most commonly used biospecimens for medical research and plays a major role in the search for new biomarkers of metabolic disorders, infectious diseases and even complex diseases such as cancer. For biobanking purposes, blood and its fractions can be readily obtained and is easily handled and stored. Plasma is effectively an abundant source of specific molecules including bioactive lipids, cell-free DNA, mRNA, non-coding RNA and soluble proteins [1–5].

Extracellular vesicles (EVs) isolated from plasma are a promising source of biomarkers both for the detection of diseases and for therapeutic drug delivery [6–8]. However, the techniques used to isolate EVs from long-term biobank-stored plasma samples have not yet been standardized [4, 9, 10].

In the absence of a recommended isolation technique, ultracentrifugation (UC) is the classic method on which most protocols are based [11]. This procedure separates the different particles according to their density, size, and shape, such that the larger denser particles will firstly sediment out. Its main drawback is that EVs are mostly co-isolated as contaminant proteins may aggregate with the EV population [6, 12, 13].

Another isolation method especially designed for human clinical samples is size exclusion chromatography (SEC). For this method, a porous stationary phase is packed into the column to separate the particles based on their size by flushing the sample through it [6, 14, 15]. The resultant fractions are composed of pure EV populations but yields are low as a result of their dilution [16, 17].

Both methods have shortcomings, especially when handling the limited volumes of plasma stored in biobanks [18, 19]. Besides, the isolation of EVs from human plasma is particularly difficult due to its high viscosity and metabolite concentrations [20–23].

To overcome these limitations and improve the specificity and purity of EV isolation, recent studies have shown that a combination of methods, such as size-based purification methods like SEC and density-based enrichment methods like UC, could enhance the efficiency of EV isolation [13, 15, 20, 24].

In the present study we compared the use of three methods to isolate EVs: UC, SEC and SEC + UC. The resultant isolates were then characterized in terms of particle size by transmission electron microscopy (TEM) and concentration by nanoparticle tracking analysis (NTA). Protein markers were detected by western blotting and the protein contents of the different isolates were determined by reverse phase-liquid chromatography coupled to high-resolution mass spectrometry (RP-LC-MS/MS).

## Materials and Methods

The aim of this study was to identify an optimal method of isolating EVs from plasma samples long-term stored in biobanks for use in investigations designed to detect new protein biomarkers.

### Plasma Samples

Samples (10 mL) of whole blood were collected from 10 healthy donors in heparin tubes and allowed to sit overnight at room temperature. The plasma was then transferred to a clean tube and stored for one year at -80ºC until use in the Collection for Leishmaniasis Research at the Spanish National Biobank Register, Ref. number C.0000898 (Royal Decree Act 1716/2011, 18th November).

### Extracellular Vesicle Isolation from Human Plasma

#### Pre-treatment of Human Plasma Samples

500 µL from each donor were thawed on ice and then centrifuged at 300 xg for 10 min at 4ºC. The supernatant was then diluted with an equal volume of filtered PBS (Thermo Scientific, Waltham, MA, USA) to reduce viscosity and centrifuged for 30 min at 2,000 xg to pellet cells and cell debris followed by further centrifugation at 12,000 xg for 30 min to pellet small debris and larger vesicles. Resultant supernatants (500 µL) from each donor (*n* = 10) were subjected to every EV isolation method in order to include inter-individual variability and further create reproducible pooled samples.

#### Ultracentrifugation (UC)

Each pre-treated plasma sample were transferred to a 4 mL ultracentrifugation tube and centrifuged at 100,000 xg for 2 h 15 min at 4ºC in a Beckman Coulter Optima XPN-100 ultracentrifuge with a SW60Ti swinging-bucket rotor (Beckman Coulter Inc, CA, USA). Following the removal of the supernatant, the pellet was resuspended in 4 mL of filtered PBS, and the ultracentrifugation step repeated. After removing the supernatant, the isolated EVs were resuspended in filtered PBS to give a final volume of 400 µL.

#### Size Exclusion Chromatography (SEC)

Following the manufacturer’s protocol, pre-treated plasma sample were placed on a 70 nm/qEV size exclusion column (Izon Science, Christchurch, New Zealand) and the flow through collected in 500 µL fractions. According to the manufacturer, EVs are eluted in Fractions 6–9 so these fractions were subjected to NTA and bicinchoninic acid assay (BCA) analysis for validation, and then combined in a final volume of 400 µL.

#### Size Exclusion Chromatography + Ultracentrifugation (SEC + UC)

Pre-treated plasma samples were first placed on the 70 nm/qEV size exclusion column and the flow through collected in 500 µL fractions. Fractions 6–9 (2 mL volume) were pelleted via two steps of ultracentrifugation at 100,000 xg for 2 h 15 min at 4ºC in a swinging-bucket rotor (SW60Ti). After ultracentrifugation, the supernatant was discarded, and the pellet re-suspended in filtered PBS to a volume of 400 µL.

### Extracellular Vesicle Characterization

Equal amounts of the 10 individual samples were combined to create pool samples for each isolation method and were characterized in duplicate by the techniques described below (MISEV2023 guidelines). For proteomic analysis, technical duplicates were performed for each isolation method.

#### Nanoparticle Tracking Analysis (NTA)

Particles purified via UC, SEC or SEC + UC were characterized using a NanoSight NS300 instrument (Malvern, Worcestershire, UK) equipped with NTA 3.2 software in terms of their concentration, mean size, and size distribution profile. For this analysis, samples were diluted 1:50 (SEC; SEC + UC) or 1:100 (UC) in PBS and continuously infused through an automatic syringe pump at a flow rate of 50 µL/min. All samples were measured using the same instrument settings: camera level 12, auto background subtraction/blur/minimum track length acquisition time 60 s, and detection threshold 5. Final concentrations were multiplied by the dilution factor.

#### Protein Quantification, SDS-page and Western Blotting

EV samples from each isolation method were lyophilized to ensure uniform protein quantity in each pool. The lyophilized samples were rehydrated with milliQ water to achieve a minimum concentration of 0.2 µg/µL for subsequent analysis.

Protein concentrations were determined by the BCA method using a Protein Assay Reagent Kit (Thermo Scientific, Waltham, MA, USA) performed according to the manufacturer’s instructions. Briefly, 10 µL of sample were used in a final reaction volume of 200 µL of BCA Working Reagent. The reaction mixture was incubated for 30 min at 37ºC. Absorbance was measured at 562 nm. Protein concentrations were calculated using bovine serum albumin (BSA) standards (Thermo Scientific, Waltham, MA, USA) and a four-parameter logistic curve.

Equal amounts of proteins (10 µg) for each isolation method were separated on a 12% SDS-PAGE gel (0.75 mm-thick, 4% stacking, and 12% resolving) and Coomasie blue stained (Merck Millipore, Billerica, MA, USA).

Total EV proteins were lysed in a loading buffer that was reducing (Tris-HCl pH 6.8 0.125 M; SDS 4%; glycerol 20%; 2-mercaptoethanol 10%; EDTA pH8 15 mM and Bromophenol blue 0.03%) or non-reducing (components as for the reducing buffer but lacking 2-mercaptoethanol) and subjected to 3 cycles of alternating hot-cold temperature every 5 min (5 min at 95ºC and 5 min on ice). Next, samples were separated on a 12% SDS-PAGE gel and transferred to a Protean® nitrocellulose blotting membrane (Amersham, GE Healthcare, Munich, Germany). Membranes were blocked in 5% non-fat milk in PBS containing 0.05% Tween-20 (PBST) (Sigma Aldrich, Merck KGaA, Darmstadt, Germany) for 1 h at room temperature. They were then incubated overnight at 4ºC with gently rocking with the primary antibodies mouse monoclonal anti-human CD81 (5A6) HRP-conjugated (Santa Cruz Biotech; sc-23,962; 1:200) and mouse monoclonal anti-human CD63 (HansaBiomed; HBM-CD63; 1:1000) for non-reducing conditions, or anti-TSG101 antibody (4A19) (abcam; ab83; 1:1000), anti-albumin (F-10) (Santa Cruz Biotech; sc-271,605; 1:500), and anti-apoA1 (Invitrogen; MA5-14667; 1:500) for reducing conditions. Membranes were washed and HRP-conjugated goat anti-mouse IgG (H + L) antibody (G21040) (ThermoFisher Scientific; G-21,040; 1:10,000) added for 2 h at room temperature for CD63, TSG101, albumin and apoA1 detection. Blots were washed with PBST, and the signal recorded using the kit ECL Pierce™ (Thermo Scientific, Waltham, MA, USA) in an Amersham ImageQuant 800 instrument (Cytiva, MA, USA).

#### Transmission Electron Microscopy (TEM)

For TEM, EV samples were diluted 1:10 in PBS and fixed with a final concentration of 2% paraformaldehyde for 5 min. Samples were then transferred to glow-discharged carbon-coated copper grids for 5 min and washed twice in MilliQ water and negatively stained with 2% aqueous uranyl acetate for 1 min. EV particles were visualized using a FEI Tecnai 12 electron microscope equipped with a LaB6 filament operated at 120 kV. Images were captured with an FEI Ceta digital camera at a nominal magnification of 30,000×.

### Proteomic Analysis

#### Protein In-Gel Digestion

For In-Gel digestion, the lyophilized protein extracts were suspended in up to 40 µL of sample buffer, and then transferred to the 1.2 cm-wide wells of a conventional SDS-PAGE gel (0.75 mm-thick, 4% stacking, 10% resolving). The run was stopped as soon as the front entered 3 mm into the resolving gel, so that the whole proteome became concentrated at the stacking/resolving gel interface. Unseparated protein bands were visualized by Coomassie staining, excised, cut into cubes (2 × 2 mm), and placed in 0.5 mL microcentrifuge tubes [25]. The gel pieces were destained in acetonitrile: water (ACN: H2O, 1:1), reduced and alkylated (disulfide bonds from cysteinyl residues were reduced with 10 mM Dithiothreitol (DTT) for 1 h at 56ºC, and thiol groups were then alkylated with 10 mM iodoacetamide for 30 min at room temperature in the dark) and digested in situ with sequencing grade trypsin (Promega, Madison, WI) as described by Shevchenko et al. [26]. with minor modifications. The gel pieces were shrunk by removing all liquid using sufficient Acetonitrile (ACN). ACN was pipetted out and the gel pieces were dried in a speedvac. The dried gel pieces were re-swollen in 100 mM Tris-HCl pH 8, 10 mM CaCl_2_ with 60 ng/µL trypsin at a 5:1 protein: enzyme (w/w) ratio. The tubes were kept on ice for 2 h and incubated at 37 °C for 12 h. Digestion was stopped by the addition of 1% trifluoroacetic acid (TFA). Whole supernatants were dried down and then desalted onto ZipTip C18 pipette tips (Millipore) until mass spectrometry analysis.

#### Reverse Phase-liquid Chromatography RP-LC-MS/MS Analysis (Dynamic Exclusion mode)

The desalted protein digest was dried, resuspended in 10 µL of 0.1% formic acid and subjected to RP-LC-MS/MS in an Easy-nLC 1200 system coupled to an ion trap LTQ-Orbitrap-Velos-Pro hybrid mass spectrometer (Thermo Scientific). Peptides were concentrated (on-line) by reverse phase chromatography using a 0.1 mm × 20 mm C18 RP precolumn (Thermo Scientific), and then separated using a 0.075 mm x 250 mm C18 RP column (Phenomenex) operating at 0.25 µL/min. Resultant peptides were eluted using a 90-min dual gradient. The gradient profile was set as follows: 5 − 25% solvent B for 68 min, 25 − 40% solvent B for 22 min, 40 − 100% solvent B for 2 min and 100% solvent B for 18 min (Solvent A: 0.1% formic acid in water, solvent B: 0.1% formic acid, 80% ACN in water). Electrospray ionization (ESI) was performed using a nano-bore emitters Stainless Steel ID 30 μm (Proxeon) interface at 2.1 kV spray voltage with 60% S-Lens. Orbitrap resolution was set at 30,000 [27].

Peptides were detected in survey scans from 400 to 1600 amu (1 µscan), followed by twenty data-dependent MS/MS scans (Top 20) using an isolation width of 2 u (in mass-to-charge ratio units), normalized collision energy of 35%, and dynamic exclusion applied at 60 s intervals. Charge-state screening was enabled to reject unassigned and singly-charged protonated ions.

### Data Analysis

For peptide identification from raw data, we used a PEAKS Studio XPro search engine (Bioinformatics Solutions Inc., Waterloo, Ontario, Canada). The database search was performed against uniprot-Homo sapiens (79,684 entries; UniProt release 06/22) (decoy-fusion database). The following constraints were used for the searches: tryptic cleavage after Arg and Lys (semispecific), up to two missed cleavage sites, and tolerances of 20 ppm for precursor ions and 0.6 Da for MS/MS fragment ions. Searches were performed allowing optional Met oxidation and Cys carbamidomethylation. False discovery rates (FDR) for peptide spectrum matches (PSM) and for protein were limited to 0.01. Only proteins with at least two unique peptides detected by RP-LC-MS/MS analysis were considered reliably identified.

The proteins identified by RP-LC-MS/MS in the EVs derived from healthy donors by different isolation methods were subjected to functional annotation with DAVID 2021 [28, 29]. This method identifies the gene ontology (GO) cellular components associated with the proteins, assigning *p*-values (Fisher’s exact test) and Benjamini-corrected *p*-values. Only strongly enriched annotation categories (Benjamini-corrected *p* < 0.01) were considered.

### Statistical Analysis

All statistical analyses were performed using the package GraphPad Prism version 9.0 (GraphPad Software Inc, CA, USA). Descriptive statistics were computed for each isolation method. Differences between groups, or methods, were assessed by a one-way analysis of variance (ANOVA) through Tukey’s honestly significant difference (HSD) multiple comparison post hoc test. Significance was set at *p* < 0.05 (*p < 0.05, **p < 0.01, ***p < 0.001).

## Results

### Characterization of Isolated EVs

**Fig. 1:**
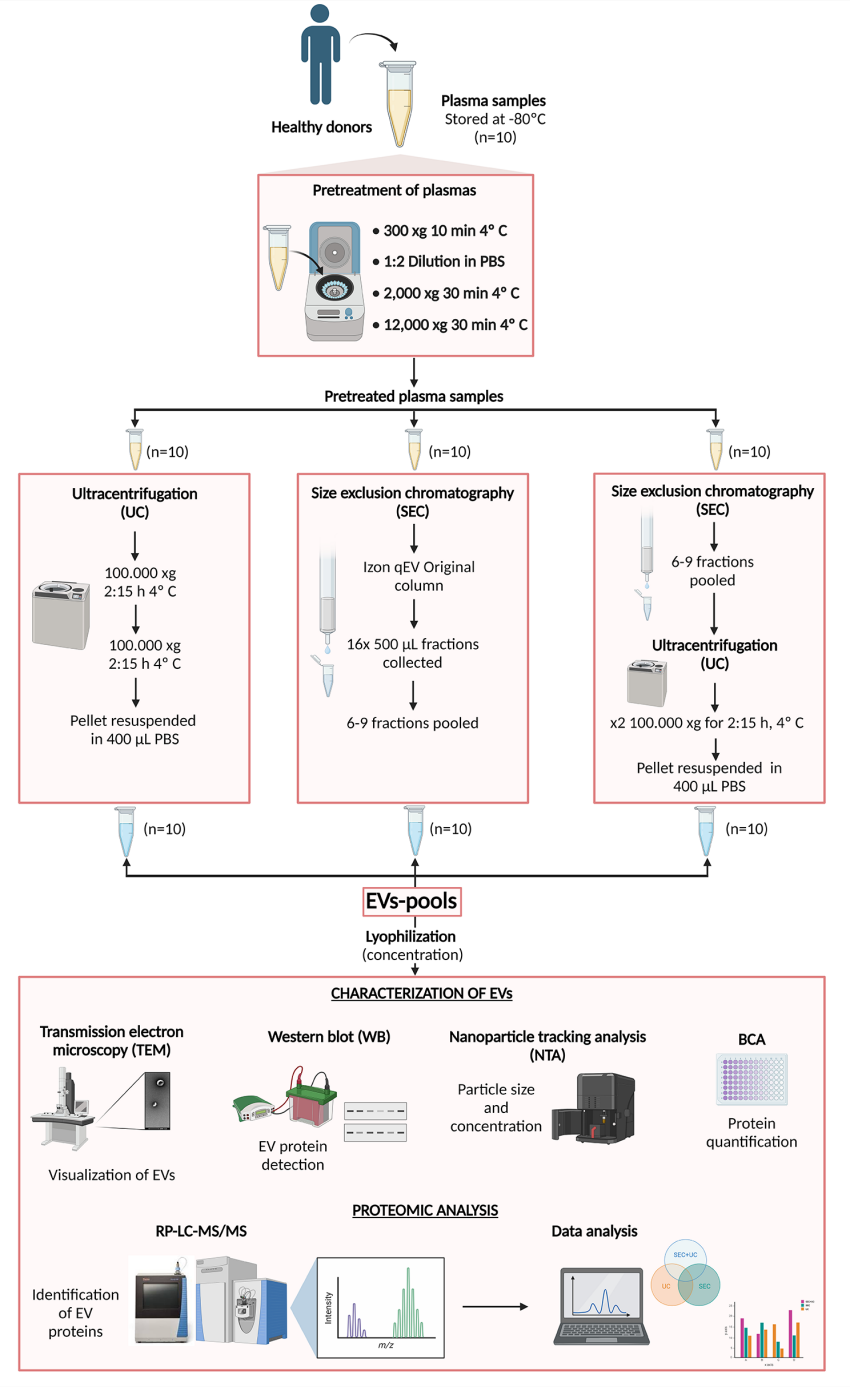
Steps of the different methods used to isolate and characterize EVs. Created with BioRender.com

The three methods of isolating EVs compared are detailed in Fig. 1.

**Fig. 2:**
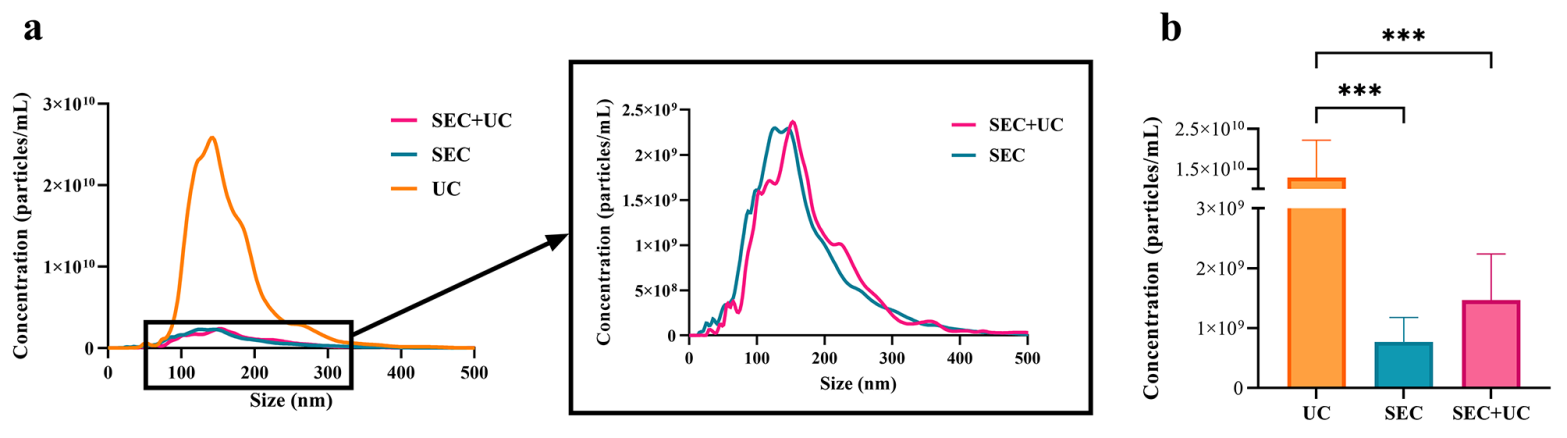
Characterization of EVs using NTA to measure (**a**) particle distributions and (**b**) total numbers of particles isolated following ultracentrifugation (UC), size exclusion chromatography (SEC), or both (SEC + UC). ***p < 0.001. The presented data has been adjusted based on the dilution of samples utilized for NTA analysis

To confirm efficient EV isolation, particle size distributions and yields for each isolation procedure were determined by NTA (Fig. 2).

Mean particle size for the three methods was greater than 100 nm. The SEC + UC group showed the largest mean size (172.1 ± 15.9 nm; mean ± SD), followed by UC (163.8 ± 9.5 nm) and Sect. (160.2 ± 25.5 nm) (Fig. 2a). However, differences were non-significant.

Our NTA analysis revealed that the UC method gave rise to a significantly greater number of isolated particles (1.28 × 10^10^ ± 9.31 × 10^9^ particles/mL; data ± SD) than Sect. (1.56 × 10^9^ ± 4.08 × 10^8^ particles/mL) or SEC + UC (1.47 × 10^9^ ± 7.73 × 10^8^ particles/mL) (*p* < 0.0001) (Fig. 2b), which failed to differ between each other.

**Fig. 3:**
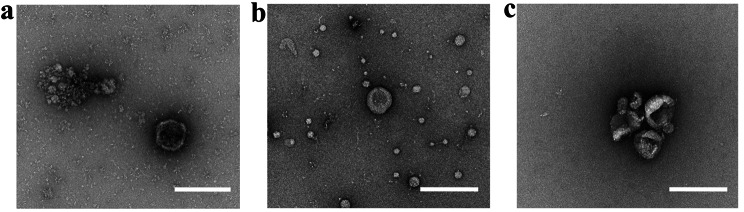
Electron microscopy images of EVs yielded by the different isolation methods. **(a)** ultracentrifugation (UC), **(b)** size exclusion chromatography (SEC), and **(c)** SEC + UC. Scale bar = 200 nm

Through TEM, we observed that all isolation procedures were successful in isolated EVs within the expected size range. Accordingly, all three methods yielded EV-like structures of characteristic cup-shaped appearance and heterogeneous sizes ranging from approximately 100–200 nm (Fig. 3). The microscopy image of EVs-UC (Fig. 3a) shows a blurry background with aggregates, indicating the co-isolation of other products. Also, the EVs-SEC image reveals the presence of rounded white vesicles resembling EVs, but these appear too small so they could be lipoprotein particles (Fig. 3b). For the combined method (Fig. 3c), a clean background may be observed with different sized EVs aggregated together as a consequence of ultracentrifugation.

**Fig. 4:**
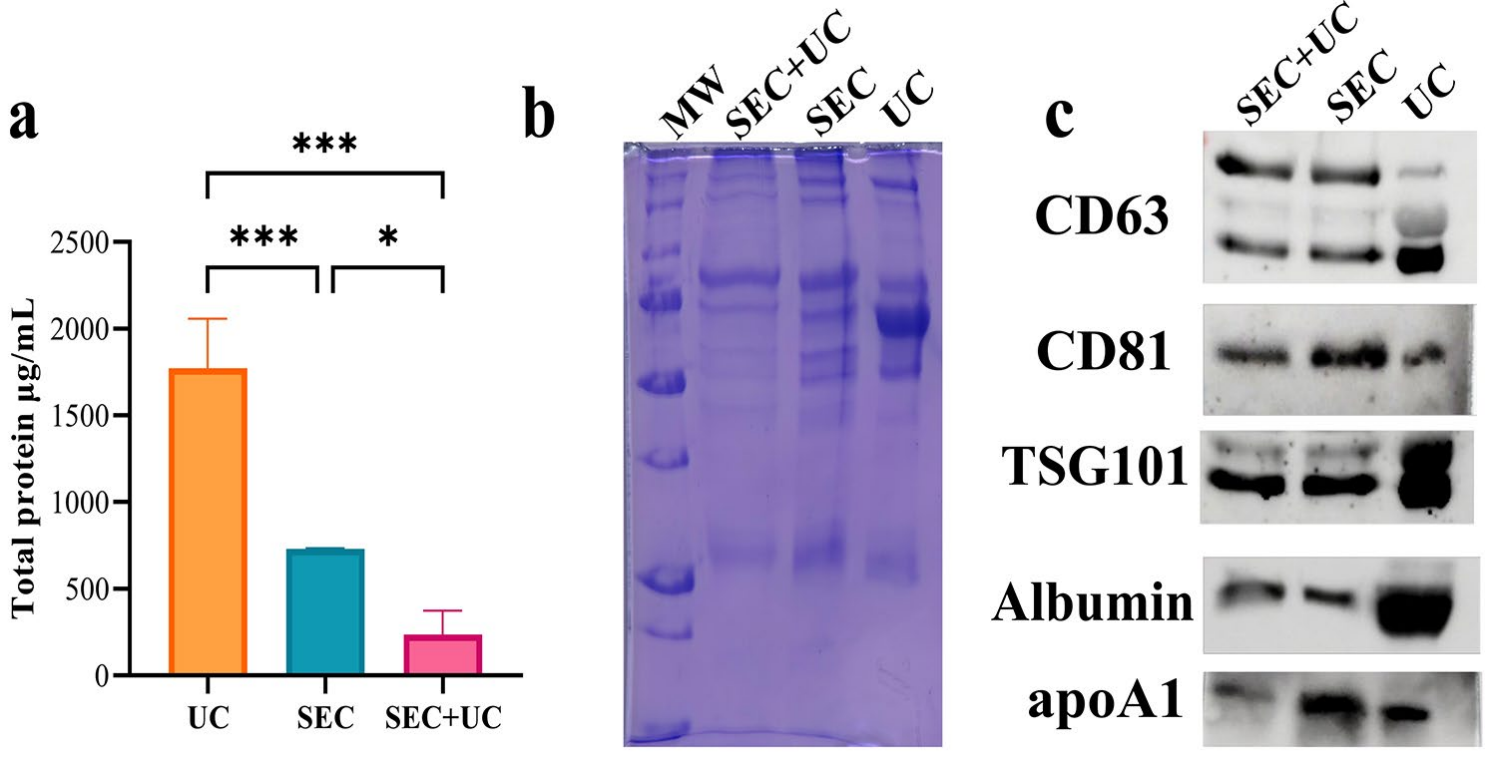
Purity of EVs isolated using different methods. **(a)** Protein concentrations of intact isolated EVs were determined by the bicinchoninic acid (BCA) assay. **(b)** Coomasie staining gel of proteins according to the EV isolation method used. **(c)** Western blotting of EVs isolated using the different methods. Data represent means ± SD of three independent experiments. *p < 0.05; ***p < 0.001

EV isolates were lysed and total protein contents (EVs plus soluble protein) of each sample were determined by BCA (Fig. 4a). Protein contents varied significantly according to the isolation method, with highest concentrations recorded for the UC group (1770.83 ± 286.4 µg/mL; mean ± SD) followed by SEC and SEC + UC (730.85 ± 4.04 and 236.77 ± 137.7 µg/mL, respectively), the combined approach yielding the lowest protein contents (*p* < 0.001).

To assess isolation efficiency, equal protein amounts in the UC-, SEC-, and SEC + UC-derived samples were placed on an SDS-PAGE gel followed by a Coomasie blue staining. As shown in Fig. 4b, there was great qualitative variation in protein patterns among the different preparations. Compared to the other EV fractions, EVs-UC were especially rich in high molecular weight proteins like immunoglobulins and albumin. Also, according to the different pattern of protein bands observed in SEC- and SEC + UC-derived EVs compared with UC-derived EVs, these two groups may have a similar protein composition (Fig. 4b).

Western blotting of the purified EV fractions confirmed the presence of the EV marker proteins CD63, CD81 (tetraspanins) and TSG101 (cytosolic protein) in 10 µg of the protein sample derived from each isolation method. As shown in Fig. 4c, all methods recovered clean EV populations. We also assessed the expression of albumin in all the EV preparations, as this marker serves to detect impurities as it is the most abundant protein in plasma. The expression of this protein was higher in EVs isolated by UC compared to SEC or SEC + UC. Additionally, we assessed lipoprotein contamination through the expression of apoA1, which was higher in both SEC and UC methods (Fig. 4c).

### MS/MS Proteomics

As plasma is a complex fluid, all MS-based proteomic procedures were conducted in duplicate samples to identify and validate as many proteins as possible. In total, 542 proteins were identified along with at least two unique peptides for all EVs derived from the different isolation methods.

The protein contents recorded for each isolation protocol revealed that the SEC + UC method yielded a more complex proteome including 364 identified proteins, compared to 212 proteins for UC and 276 proteins for SEC. These proteins were further analysed using the DAVID database, mapping them only for cellular component (CC) against the human genome as background to determine their associations with extracellular vesicles and plasma. The identification and classification of these proteins within the GO terms ‘extracellular exosome’ and ‘blood microparticle’ are provided in Additional Table 1. This analysis revealed that the isolation method SEC + UC gave rise to more EV proteins (273 proteins) and fewer blood-related proteins (70 proteins) compared to UC, which showed the largest number of plasma proteins (153 EV proteins vs. 116 plasma proteins), or SEC. (200 EV proteins vs. 92 plasma proteins).

We then conducted a more thorough assessment to determine whether commonly enriched terms were more associated with EVs or plasma across the isolation methods. The terms related to EVs examined were ‘extracellular exosome’ (GO: 0070062), ‘extracellular space’ (GO: 0005615), ‘extracellular region’ (GO: 0005576), ‘vesicle’ (GO: 0031982) and ‘extracellular vesicle’ (GO: 1,903,561). The plasma related terms considered were ‘plasma membrane’ (GO: 0005886) and ‘blood microparticle’ (GO: 0072562). Out of a total of 364 proteins identified for SEC + UC, 276 for SEC and 212 for UC, the DAVID database recognized 256, 195 and 157 gene entries, respectively.

Table 1 lists the GO terms associated with EV and plasma proteins. Terms related to EVs consistently showed comparable percentages among the different EV samples for the term ‘extracellular exosome’ with 76.2% for SEC + UC, 75.4% SEC and 75.2% UC or ‘extracellular vesicle’ with 3.9% in SEC + UC, 3.6% in SEC and 3.2% in UC. For the terms ‘extracellular space’, ‘extracellular region’, and ‘vesicle’ differences in the percentages were found in the different sample. However, for the plasma-related terms, especially ‘blood microparticle’ a higher percentage of gene entries was recorded in the UC group (21.1%, 33.3%, and 52.9% of identified proteins in SEC + UC, SEC and UC, respectively). No differences among the various methods were recorded for ‘plasma membrane’.

**Table 1:** Cellular component analysis (GO terms) of proteins related to extracellular vesicles or plasma for each of the isolation methods

		SEC + UC				SEC				UC				
Term	GO term	Count	%	*P-Value*	Benjamini	Count	%	*P-Value*	Benjamini	Count	%	*P-Value*	Benjamini	
Extracellular exosome	GO:0070062	195	76.2	2.60E-134	7.99E-131	147	75.4	2.80E-100	9.10E-98	118	75.2	3.20E-80		2.60E-78
Extracellular space	GO:0005615	123	48	2.40E-56	3.10E-54	105	53.8	1.40E-54	1.50E-52	117	74.5	1.00E-84		1.30E-82
Extracellular region	GO:0005576	115	44.9	2.50E-44	2.40E-42	106	54.4	3.00E-51	2.50E-49	113	72	5.50E-74		3.30E-72
Vesicle	GO:0031982	20	7.8	9.4E-13	2.2E-11	15	7.7	1.50E-09	2.60E-08	7	4.5	2.30E-03		1.20E-02
Extracellular vesicle	GO:1,903,561	10	3.9	1.40E-04	1.10E-03	7	3.6	3.50E-03	2.00E-02	5	3.2	3.00E-02		1.20E-01
Plasma membrane	GO:0005886	120	46.9	2.40E-12	5.40E-11	96	49.2	9.20E-12	3.40E-10	81	51.6	1.90E-11		3.30E-10
Blood microparticle	GO:0072562	54	21.1	5.20E-64	9.80E-62	65	33.3	3.80E-93	6.30E-91	83	52.9	1.50E-143		3.60E-141

UC: ultracentrifugation; SEC: size exclusion chromatography; SEC + UC: size exclusion chromatography + ultracentrifugation. *p*-values and Benjamini score distributions of DAVID predicted gene ontology and enrichment analysis terms are shown.

**Fig. 5:**
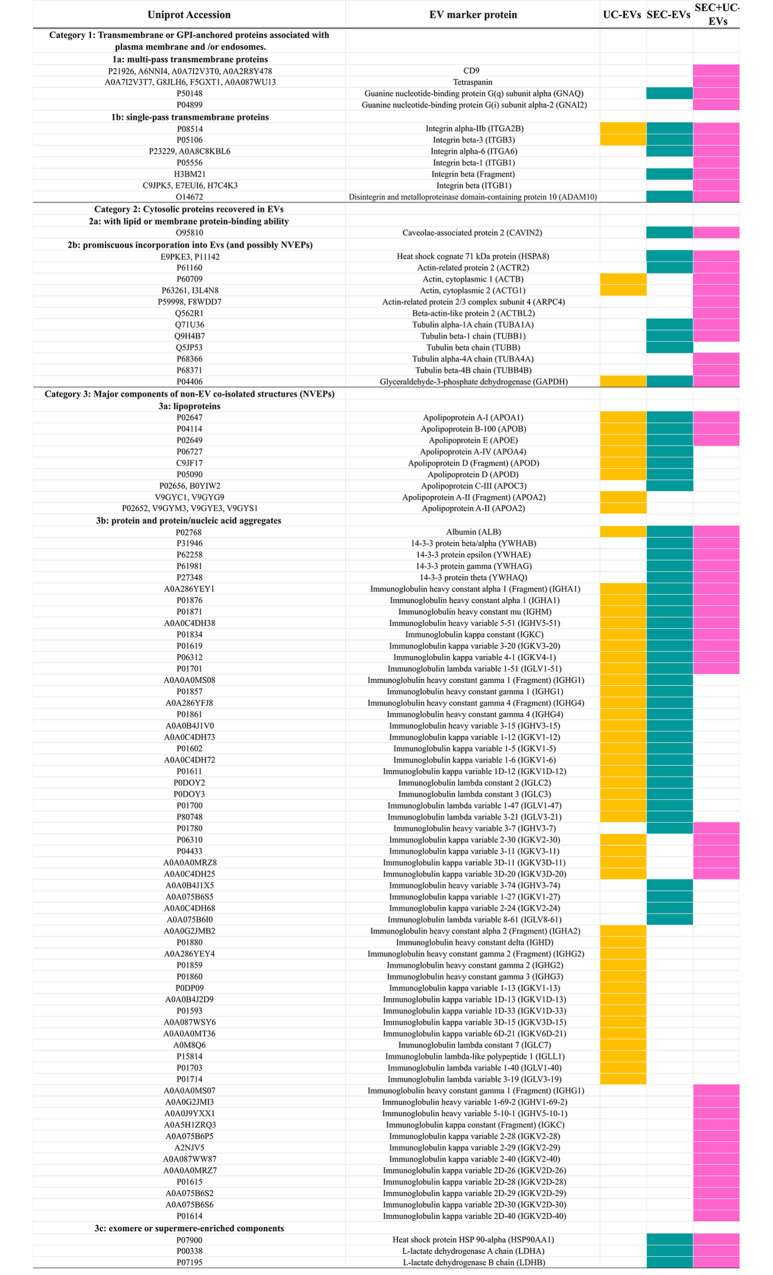
Characterization of EV protein contents based on MISEV2023 guidelines. Each row represents the identified protein in the samples within the different categories, and columns indicate the isolation method in which the protein was detected

Finally, we conducted a detailed analysis to validate the presence of proteins related to EVs and potential contaminants in the samples examined previously according to MISEV2023 guidelines from ISEV [20]. The protein samples shown in Fig. 5 were classified into three distinct categories of markers. Categories 1 and 2 indicate the detection of EVs with traditional EV markers such as integrins (Uniprot: ITG), actins (Uniprot: ACTB), or glyceraldehyde-3-phosphate dehydrogenase (Uniprot: GAPDH) in all samples. Additionally, heat shock protein 71KDa (Uniprot: (HSPA8), tetraspanins (Uniprot: CD9), guanine nucleotide (Uniprot: GNA), disintegrins (Uniprot: ADAM10), tubulins (Uniprot: TUB) or caveolae-associated protein 2 (Uniprot: CAVIN2) were found in the SEC + UC and SEC sample groups. However, the UC sample showed the least identification of EV protein markers.

Category 3 indicates the presence of common contaminants for purity assessment. As plasma samples were used, protein impurities from plasma such as albumin (Uniprot: ALB), apolipoproteins (Uniprot: APO), immunoglobulins, and others were found in all isolated EVs, but particularly in UC-EVs. A notable observation is that the number of apolipoproteins, common protein contaminants plasma-derived EVs, found in SEC + UC-EVs were clearly reduced compared to single-step techniques. Furthermore, proteins like 14-3-3 beta/alpha (Uniprot: YWHAH), heat shock protein 90 alpha (Uniprot: HSP90AA1) or lactate dehydrogenase (Uniprot: LDH) were exclusively identified in SEC and SEC + UC-EVs (Fig. 5).

**Fig. 6:**
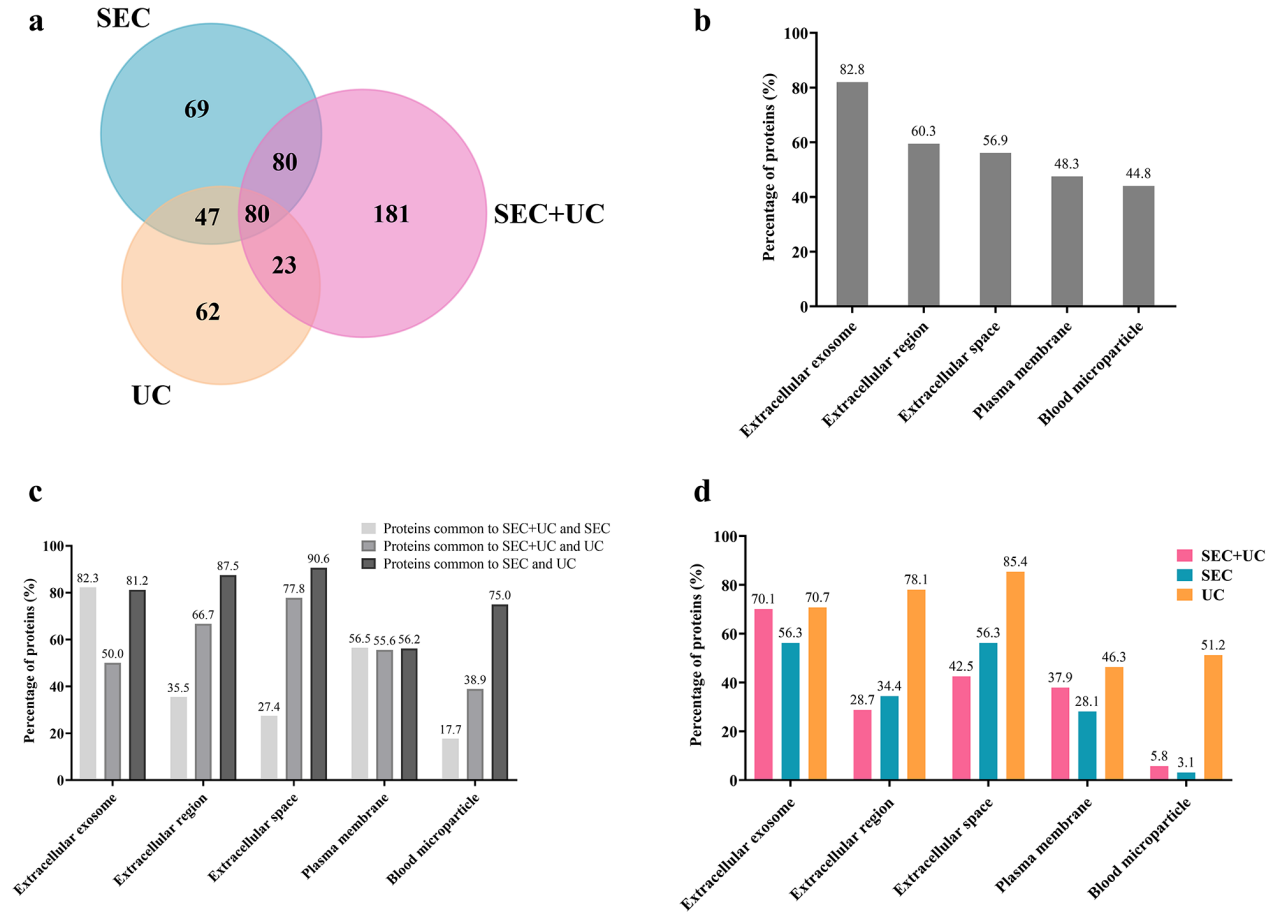
Total protein contents of EV samples yielded by the three isolation methods. **(a)** Venn diagram showing the proteins identified according to the isolation method. **(b)** Percentages of shared cellular component terms of EVs and plasma: among the 80 proteins common to all three methods; **(c)** among the proteins common to pairs of isolation methods; and **(d)** among the proteins unique to each method

After our initial assessment of EV and plasma proteins across all samples, we performed a comparative analysis to identify shared and unique proteins in the different isolation groups. Overlap in protein identification was visualized using a Venn Diagram generated with the FunRich tool (Fig. 6a).

In all three isolation method groups, 80 proteins were found to be common. Our GO enrichment CC analysis revealed 5 enriched terms related to EVs or plasma among these shared proteins as detailed in Fig. 6b. GO classifications indicated 82.8% of these proteins were categorized as ‘extracellular exosomes’. Furthermore, 60.3% were linked to the term ‘extracellular region’, and 56.9% to ‘extracellular space’. These percentages were higher than the proportion of plasma-related proteins (‘blood microparticle’) detected (44.8%).

Upon closer examination of overlapping proteins across groups (Fig. 6a), it becomes evident that the method pairs SEC + UC and SEC gave rise to a higher number of shared proteins (80) than SEC and UC (47) or SEC + UC and UC [23].

In a more detailed analysis, we examined GO terms related to EVs and to plasma within proteins common to the different isolation methods compared as pairs.

As may be observed in Fig. 6c, at the general level, terms related to EVs appear in greater proportions than the plasma-related terms in all comparisons. In effect, proteins common to SEC and UC showed the highest proportions of the terms ‘extracellular exosome’, ‘extracellular region’ and ‘extracellular space’, at 81.2%, 87.5% and 90.6%, respectively, compared to the pairs SEC + UC and UC, and SEC + UC and SEC. However, it should be noted that all isolation methods yielding proteins common with UC (SEC + UC and UC, and SEC and UC) gave rise to greater proportions of the plasma-related term ‘blood microparticle’, at 75% for SEC and UC, and 38.9% for SEC + UC and UC.

Once we had established proteins shared among the different isolation methods, we focused our next analysis on identifying proteins exclusive to each isolation method. The Venn diagram in Fig. 6a reveals that 181 proteins were exclusive to EVs isolated from plasma using the SEC + UC method, 62 proteins were exclusive to the single-step UC method, and 69 proteins were exclusive to the SEC method.

We then annotated unique proteins arising from each isolation method by assessing enriched GO terms for CC and comparing the top 5 enriched GO terms shared by the different isolation method groups.

Remarkably, according to the data presented in Fig. 6d, all unique proteins showed a marked abundance of GO terms associated with EVs such as ‘extracellular space’, ‘extracellular region’, and ‘extracellular exosome’. This suggests that a great majority of these unique proteins can be attributed to EVs. Interestingly though, among proteins exclusive to UC, high proportions of enrichment in plasma-related terms were detected (‘blood microparticle’ and ‘plasma membrane’ at 51.2% and 46.3%, respectively). This pattern was not apparent for the other two methods.

Considering that most unique proteins could be associated with EVs, we then focused on these unique proteins as a measure of the sensitivity of the different isolation methods and also tried to determine whether any important information could be missing according to the isolation protocol used. To this end, we took as reference values theoretical concentrations of these unique proteins in plasma as defined in the Human Protein Atlas database (https://www.proteinatlas.org/ accessed on November 23, 2023). These data are provided in Table 2 and in Additional Table 2.

**Table 2:** Ranges of unique proteins identified for each isolation method

	UC			SEC			SEC + UC		
	mg/L	µg/L	ng/L	mg/L	µg/L	ng/L	mg/L	µg/L	ng/L
Range	440 − 1.2	730 − 4.5	NI	45 − 1.4	840 − 6.4	870 − 7.1	300 − 1.1	830 − 1.1	940 − 150
Percentage of proteins (%)	87	12.24	0	23	58	17	9	81	9.01

UC: ultracentrifugation; SEC: size exclusion chromatography; NI: not identified.

The dynamic ranges of the unique proteins identified for each isolation method were categorized by concentration rates (mg/L, µg/L, and ng/L). The percentages shown in the table indicate the proportions of proteins within each specified concentration range detected for each of the three isolation methods.

The SEC + UC method emerged more effective for the identification of different proteins across various concentration ranges. The highest detection rate, 81% of unique proteins, was observed within the µg/L range, followed by 9.01% and 9% in the ng/L and mg/L ranges respectively. Protein isolation using SEC followed a similar trend with higher detection rates of 58% and 17%, respectively for µg/L and ng/L concentrations compared to 23% for mg/L concentrations. Although SEC method was able to identify proteins with the lowest theoretical concentration in plasma (7.1 ng/L), SEC + UC detected a higher number of proteins within this concentration when compared to SEC (11 proteins vs. 8 proteins) (Additional Table 2).

Notably, in the isolates produced by UC, it was possible to identify distinct proteins present in plasma at mg/L concentrations (87%), whereas only six proteins detected were within the µg/L range.

## Discussion

Extracellular vesicles (EVs) have recently gained special attention in the field of biomarkers, mostly because of their possible applications for the diagnosis of various diseases and for monitoring disease progression and treatment efficacy [7, 30, 31]. While plasma samples are useful for large-scale studies due to their availability in biobanks [23, 32], isolating EVs from these samples remains a challenge because of their limited volumes and long-term frozen storage. The present study was designed to compare the protein profiles of EVs isolated using different methods in an effort to identify the optimal method for use with plasma samples long-term stored frozen at − 80 °C.

EVs isolated from biobank-stored plasma samples are highly stable [18, 33, 34]. In effect, we found the presence of cup-shaped particles indicating that long-term storage and the different isolation methods examined here did not especially affect the morphology of the resultant EVs [35].

When we compared the three isolation methods, particle concentrations were higher in the UC group. Ultracentrifugation works as a precipitation technique, leading to the isolation of both EVs and contaminating proteins like albumin, fibrinogen and lipoproteins. Effectively, the UC method yielded higher concentrations of total proteins than SEC and SEC + UC together. As it is difficult to differentiate between EV particles and other similarly sized protein aggregates, we used several approaches to determine the efficiency of EVs isolated using the different methods including NTA, western blot analysis and TEM [5, 24, 36]. Our results indicated that among the different isolation methods, the EVs-UC isolates were especially prone to protein aggregation and to show albumin expression. To improve the purity of isolated EVs, an albumin-depletion step is usually introduced [37, 38]. However, this is not possible when handling limited plasma volumes.

The SEC method has been proposed as a possible solution for handling low sample volumes and preventing contamination by plasma proteins. However, this method has not yet been tested on long-term stored biobank plasma samples [39, 40]. The combined use of SEC with other separation methods has been recently suggested to overcome the limitations of UC and SEC alone [5, 13, 41]. Although none of the isolation methods compared here completely eliminated plasma proteins, SEC + UC and SEC preparations exhibited lower albumin expression, while only the combined method achieved the lowest apoA1 apolipoprotein expression, suggesting purer EV populations.

After this preliminary assessment of the three isolation methods, we conducted a proteomic analysis of EV cargo. In an analysis of the proteins yielded by each isolation method, we found that with the SEC + UC method it was possible identify a significantly larger number of proteins in comparison to UC and SEC alone. Additionally, when comparing proportions of EV proteins to plasma proteins, and using MISEV2023 guidelines for validating the presence of EV, it emerged that the SEC + UC method gave rise to the highest number of EV-associated proteins and a lower identification of lipoproteins, which are commonly recognized as protein impurities in plasma-derived EVs. Conversely, in the EVs-UC isolates, we observed greater numbers of contaminating plasma-related proteins, which could impair the detection of EV proteins. Although a similar limitation has been described for SEC when using low sample volumes [24, 40], its use in combination with UC has been reported to improve the proteomic characterization of EVs in fresh plasma samples [31, 41]. Using this combined approach, we obtained similar results in our samples of human plasma stored frozen in biobanks for one year. Our findings are proof of concept of the efficiency of the SEC + UC EV isolation method for use in proteomic studies.

Our analysis of proteins common to pairs of different isolation methods revealed that pairs including the UC group showed higher proportions of plasma proteins. The combination approach yielded a substantial number of unique proteins in comparison with the single-step methods. These unique proteins were predominantly associated with EVs based on cellular component annotations. Moreover, a significant portion of these identified proteins were noted to be found, albeit theoretically, in low concentrations in human plasma. This observation suggests that the combined method successfully identify a higher number of low-abundance proteins, indicating increased sensitivity compared to the other methods.

A few studies have consistently shown that the use of a combination of two or more EV isolation methods results in the enhanced identification of EVs markers. In these reports, EVs were successfully isolated from plasma, improving the proteomic profiling of extracellular vesicles [5, 35, 42]. However, in such studies, EVs were isolated from larger volumes of fresh plasma [13, 40, 43] avoiding the limitations of the use of biobank samples. The only literature studies centred on EV isolation from frozen human plasma samples did not use the SEC + UC combination [3, 42, 44] or did not involve proteomics after the use of this combined isolation method for EVs [35, 36].

Our findings suggest that this new approach enables the comprehensive detection and characterization of less abundant proteins that might be otherwise masked by plasma-derived proteins. This is particularly advantageous when targeting proteins present in low concentrations in plasma, as these may not be detected using the UC or SEC methods alone. In consequence, SEC + UC could be a promising tool for advances in the field of biomarker research [45, 46].

Our work has several limitations. As reported by others, the yield and purity of different extracellular fractions may be influenced by cell density, cell stress or drug exposure among other factors [8]. In addition, while we analysed human plasma from healthy persons stored for one year at -80ºC, other storage or health status conditions could alter the results obtained. Further work is therefore needed to explore the use of this isolation method for the discovery of protein biomarkers.

## Conclusions

The use of the combined EV isolation method (SEC + UC) resulted in the detection of more protein species by proteomics. This approach could offer valuable insights into physiological and pathological processes in studies examining EV cargo loads in frozen plasma samples from patient cohorts stored in biobanks. In addition, protein content analysis could help discover new biomarkers with diagnostic and prognostic applications.

### Electronic Supplementary Material

Below is the link to the electronic supplementary material.

Supplementary Material 1

Supplementary Material 2

## Data Availability

The dataset of the mass spectrometry proteomics have been deposited to the ProteomeXchange Consortium via the PRIDE partner repository with the dataset identifier PXD049342.

## Abbreviations

EVs: Extracellular vesicles
UC: Ultracentrifugation
SEC: Size exclusion chromatography
SEC + UC: Size exclusion chromatography + ultracentrifugation
NTA: Nanoparticle tracking analysis
BCA: Bicinchoninic acid assay
TEM: Transmission electron microscopy
SDS-PAGE: Sodium dodecyl sulfate polyacrylamide gel electrophoresis
RP-LC-MS/MS: Reversed-Phase liquid chromatography
FDR: False discovery rate
PSM: peptide spectrum matches
TSG101: Tumor susceptibility gene 101
IgG: Immunoglobulins
ACT: actin
HSP: heat shock protein
ALB: albumin
ANXA: annexins
ITG: integrins
APO: apolipoproteins
FIB: fibrinogens
HB: haemoglobins
GO: Gene Ontology
CC: Cellular component
MISEV: Minimal information for studies of extracellular vesicles
ISEV: International Society for Extracellular Vesicles
